# Supplementary material for: Chemotaxonomy of Southeast Asian Peperomia (Piperaceae) Using High-Performance Thin-Layer Chromatography Colour Scale Fingerprint Imaging and Gas Chromatography–Mass Spectrometry
Source: Plants (Basel). 2024 Sep 30;13(19):2751. doi: 10.3390/plants13192751 (PMC11478959; doi:10.3390/plants13192751)
Supplement: Supplementary file 1 [file plants-13-02751-s001.zip › plants-3161315-supplementary.pdf]

Supplement material

The HPTLC multiwavelength imaging using the ethyl acetate extract separated the 14 *Peperomia* into seven groups. These patterns can be clearly seen in both the cluster dendrogram and PCA analyses. Both analyses show very similar results. The PC1 and PC2 axes combined were able to explain 65.19% of the total variance (Figure S1).

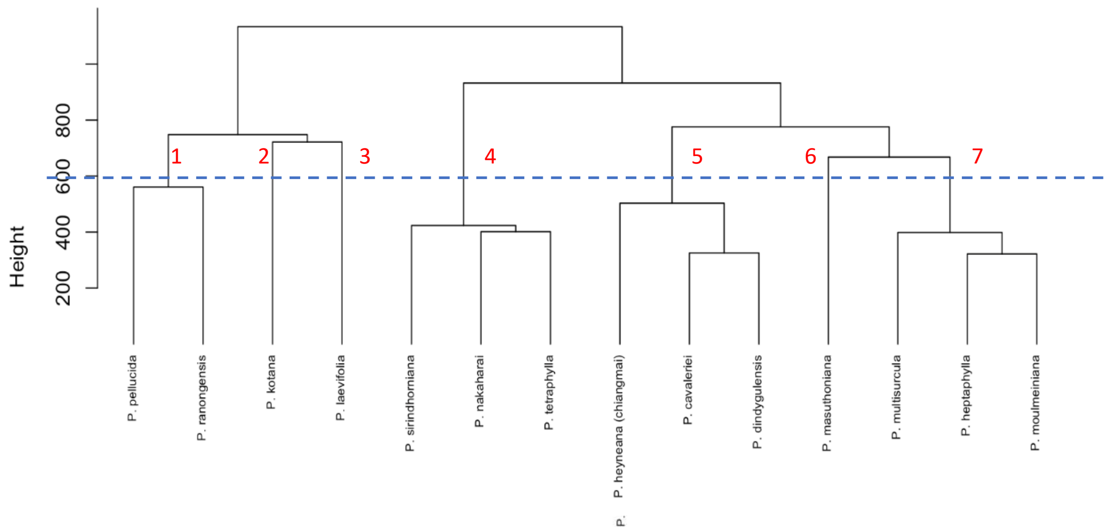

(a)

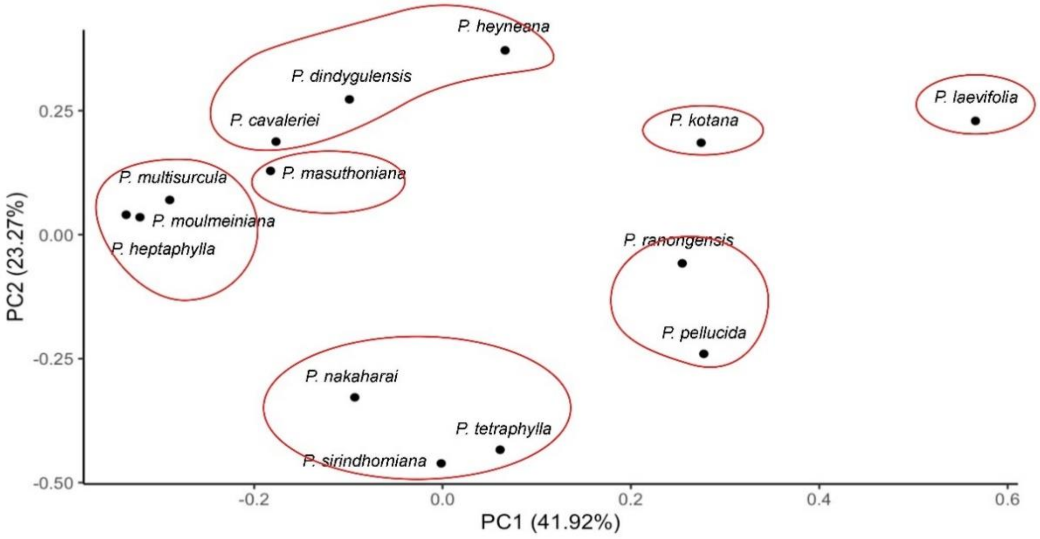

(b)

Figure S1 Clustering dendrogram and PCA of the HPTLC based on the color scale fingerprint data using the ethyl acetate extract for 14 *Peperomia* samples

The cluster dendrogram showing the hierarchical relationships of 14 species of *Peperomia,* using the ethanol extract, divided the samples into seven groups (Supplementary Figure S2). The analysis results using the cluster dendrogram and the PCA are consistent with each together. The PCA axes was able to explain 66.41% of total variance (Figure S2) in PC1 and PC2.

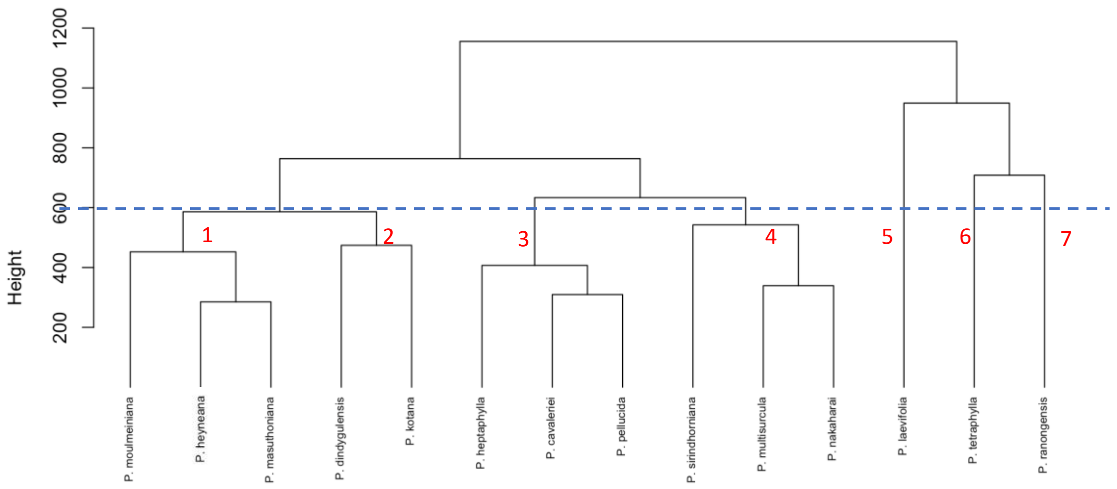

(a)

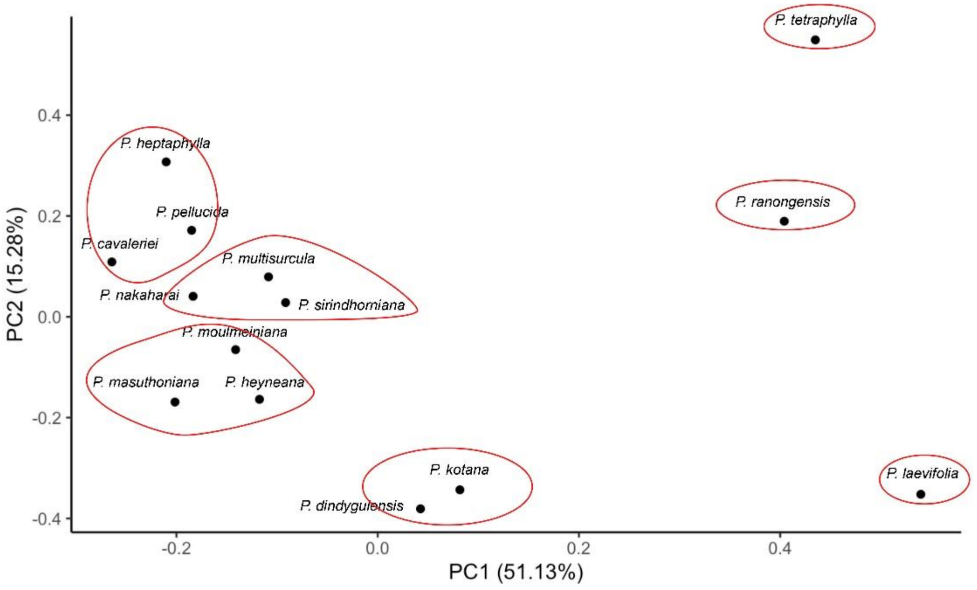

(b)

Figure S2 Clustering dendrogram and PCA profile of the HPTLC based on color scale fingerprints data, using the ethanol extract, for 14 *Peperomia* samples.

Table S1 Chemical compounds detected in each of 14 species of *Peperomia* using the haxane extract and GC-MS.

No	Compound	*Peperomia* species														Number of species found
		P. cavaleriei	*P. dindygulensis*	*P. heptaphylla*	*P. heyeana*	*P. kotana*	*P. laevifolia*	*P. masuthoniana*	*P. moulmeiniana*	*P. multisurcula*	*P. nakaharai*	*P. pellucida*	*P. sirindhorniana*	*P. tetraphylla*	*P. ranongensis*	
1	alpha.-Pinene				/			/								2
2	beta.-Pinene		/		/		/	/								4
3	Copaene			/	/										/	3
4	Caryophyllene	/						/				/		/		4
5	Humulene													/		1
6	Germacrene D		/													1
7	Pentadecane											/				1
8	beta.-Bisabolene	/														1
9	(-)-Spathulenol													/		1
10	Carotol											/				1
11	8-Heptadecene								/		/					2
12	Apiol											/				1
13	Eicosane								/		/					2
14	n-Hexadecanoic acid	/		/	/	/	/	/	/	/	/	/	/	/	/	13
15	Hexadecanoic acid, ethyl ester	/		/			/		/	/	/		/			7
16	Phytol						/								/	2
17	Linoelaidic acid	/	/	/		/	/		/	/	/	/	/		/	11
18	7-Tetradecenal, (Z)-		/	/	/	/	/		/	/	/	/		/	/	11
19	cis,cis,cis-7,10,13-Hexadecatrienal	/								/	/					3
20	Linoleic acid ethyl ester			/							/					2
21	9,12,15-Octadecatrienoic acid, (Z,Z,Z)-	/		/												2
22	9,12-Octadecadienoic acid (Z,Z)-	/					/		/							3
23	Eicosanoic acid	/														1
24	Octadecanoic acid, ethyl ester		/	/						/		/			/	5
25	(E)-1-(2-Hydroxy-4,6-dimethoxyphenyl)-3-phenylprop-2-en-1-one								/		/					2
26	Tetracosane	/						/		/	/			/		5
27	Flavone, 5-hydroxy-7,8-dimethoxy-								/		/					2
28	4H-1-Benzopyran-4-one, 5,7-dimethoxy-2-phenyl-								/		/					2

Table S1 *cont.*

No	Compound	*Peperomia* species														Number of species found
		P. cavaleriei	*P. dindygulensis*	*P. heptaphylla*	*P. heyeana*	*P. kotana*	*P. laevifolia*	*P. masuthoniana*	*P. moulmeiniana*	*P. multisurcula*	*P. nakaharai*	*P. pellucida*	*P. sirindhorniana*	*P. tetraphylla*	*P. ranongensis*	
29	Hexacosanal									/						1
30	Epicholestanol					/										1
31	Tetratetracontane	/	/	/	/		/	/	/	/	/	/	/	/	/	13
32	Octacosanal	/	/	/	/			/	/	/	/			/		9
33	Hentriacontane	/	/		/		/		/	/	/	/	/	/	/	11
34	1-nonanone, 1-(2,5-dihydroxyphenyl)-		/		/			/								3
35	(+)-Sesamin							/		/						2
36	Campesterol			/			/									2
37	Ergost-5-en-3-ol, (3.beta.)-	/				/					/	/	/	/	/	7
38	E,E-2,13-Octadecadien-1-ol							/								1
39	Stigmasterol	/	/	/		/			/	/	/	/	/	/	/	11
40	gamma.-Sitosterol	/	/	/	/	/	/	/	/	/	/	/	/	/	/	14

Table S2 Comparison of the chemical compounds recorded from this study with previous reports in *Peperomia* and *Piper* species.

No	Compound	Plant species												
		P. pellucida in this study	*P. pellucida*	*P. pellucida*	*P. pellucida*	*P.macrostachya*	*P. rotundifolia*	*P. inaequalifolia*	*P.blanda*	*P. leptostachya*	*Piper aduncum*	*Piper betle*	*Piper crocatum*	*Piper retrofractum*
1	alpha.-Pinene				/	/	/	/		/				
2	beta.-Pinene				/	/	/	/		/				
3	Copaene				/	/	/	/		/				
4	Caryophyllene	/	/	/	/	/	/	/	/	/	/	/	/	
5	Humulene				/	/	/			/				
6	Germacrene D				/					/	/	/	/	
7	Pentadecane	/			/	/	/		/					
8	beta.-Bisabolene					/	/	/				/	/	
9	(-)-Spathulenol				/	/		/		/				
10	Carotol	/	/		/									
11	8-Heptadecene*													
12	Apiol	/	/	/	/		/			/				
13	Eicosane*													
14	n-Hexadecanoic acid	/	/						/		/	/	/	/
15	Hexadecanoic acid, ethyl ester			/							/	/	/	/
16	Phytol		/	/					/		/	/	/	/
17	Linoelaidic acid*	/												
18	7-Tetradecenal, (Z)-*	/												
19	cis,cis,cis-7,10,13-Hexadecatrienal*													
20	Linoleic acid ethyl ester*													
21	9,12,15-Octadecatrienoic acid, (Z,Z,Z)-		/								/	/	/	/
22	9,12-Octadecadienoic acid (Z,Z)-		/	/							/	/	/	/
23	Eicosanoic acid*										/	/	/	/
24	Octadecanoic acid, ethyl ester*	/									/	/	/	/
25	(E)-1-(2-Hydroxy-4,6-dimethoxyphenyl)-3-phenylprop-2-en-1-one*													
26	Tetracosane*													/
27	Flavone, 5-hydroxy-7,8-dimethoxy-*													
28	4H-1-Benzopyran-4-one, 5,7-dimethoxy-2-phenyl-*													
29	Hexacosanal*										/	/		/

Table S2 *Cont.*

No	Compound	Plant species												
		P. pellucida in this study	*P. pellucida*	*P. pellucida*	*P. pellucida*	*P.macrostachya*	*P. rotundifolia*	*P. inaequalifolia*	*P.blanda*	*P. leptostachya*	*Piper adumcum*	*Piper betle*	*Piper crocatum*	*Piper retrofractum*
30	Epicholestanol*													
31	Tetratetracontane*	/									/	/	/	/
32	Octacosanal*											/		/
33	Hentriacontane*	/									/	/		/
34	1-nonanone, 1-(2,5-dihydroxyphenyl)-*													
35	(+)-Sesamin*													
36	Campesterol		/											
37	Ergost-5-en-3-ol, (3.beta.)-*	/												
38	E,E-2,13-Octadecadien-1-ol		/											
39	Stigmasterol	/	/						/					
40	gamma.-Sitosterol	/	/						/					
Solvent/Method		Hexane	ethanol	Methanol	Distillation	Distillation	Distillation	Distillation	petroleum ether, then dichloromethane and	Distillation	ethanol and chloroform	ethanol and chloroform	ethanol and chloroform	ethanol and chloroform
Library/Reference		Wiley 6, NIST02 and NIST 17	NIST 2005 library	(NIST 2017 Library; software: Open Lab	n-alkanes homologous series	n-alkanes homologous series	n-alkanes homologous series	Adams, R.P. and ,NIST Chemistry	Wiley; mass spectral library	Adams book	Metaboanalyst program	Metaboanalyst program	Metaboanalyst program	Metaboanalyst program
Reference			(1)	(2)	(3)	(3)	(3)	(4)	(5)	(6)	(7)	(7)	(7)	(7)

Table S2 Reference :

(1) Narayanamoorthi, V.; Vasantha,K.,Rency, R.C.; Maruthasalam, A. GC MS determination of bioactive components of Peperomia pellucida (L.) Kunth. *Bioscience Discovery*, **2015**, 6(2). 83-88.

(2) Babu, Y.T.R; Satapathy, P.; Krishna, C.M.; Ramalakshmana, J.; Padal, S.B. GC-MS analysis and screening of antimicrobial potentialities of the medicinal herb Peperomia pellucida (L.) Kunth. *Journal of Xidian University*, **2023**, DOI:10.37896/jxu17.8/118

(3) de Liraa, P.N.B.; da Silvaa, J.K.R.; Andradeb,E.H.A.; Sousac, P.J.C.; Silvad, N.N.S.; Maiab, J.G.S., Essential Oil Composition of Three Peperomia Species from the Amazon, Brazil. *Natural Product Communications*, **2009**,4(3), 427-430

(4) Valarezo, E.; Herrera-García, M; Astudillo-Dávila, P.; Rosales-Demera, I; Jaramillo-Fierro, X.; Cartuche, L.; Meneses, M.A.; Morocho, V. Study of the Chemical Composition and Biological Activity of the Essential Oil from Congona (Peperomia inaequalifolia Ruiz and Pav.). *Plants*, **2023**, 12, 1504. https://doi.org/ 10.3390/plants12071504

(5) Al-Madhagi, W.M.; Hashim, N.M.; Awad Ali, N.A.; Alhadi, A.A.; Halim, S.N.A.;Othman, R. Chemical profiling and biological activity of Peperomia blanda (Jacq.) Kunth. *PeerJ*, **2024**, DOI 10.7717/peerj.4839

(6) Nguyen, H.M.; Pham, T.V.; Vo, H.Q.; Nguyen, H.T.; Nguyen,L.T.K.; Nguyen, B.C.; Chung, K.L.; Ho, D.V. Essential Oil from Vietnamese Peperomia leptostachya Hook. & Arn. (Piperaceae): Chemical Composition, Antioxidant, Anti-Inflammatory, Cytotoxic Activities,and In Silico Analysis. *Molecules* **2024**, 29, 2808. https://doi.org/10.3390/molecules29122808

(7) Hartini, Y.S.; Utaminingsih, J. J; Patra-murti,C.; Nugroho L.H. Secondary metabolite profile in mature and old leaves of four piper species: Forest Betel (*Piper aduncum* L.), Red Betel (*Piper crocatum* Ruiz & Pav.), Javanese Chili Betel (*Piper retrofractum* Vahl.), and Green Betel (*Piper b*etle L.). *Plant Science Today*, **2024**, 11(2), 546-552. https://doi.org/10.14719/pst.3070

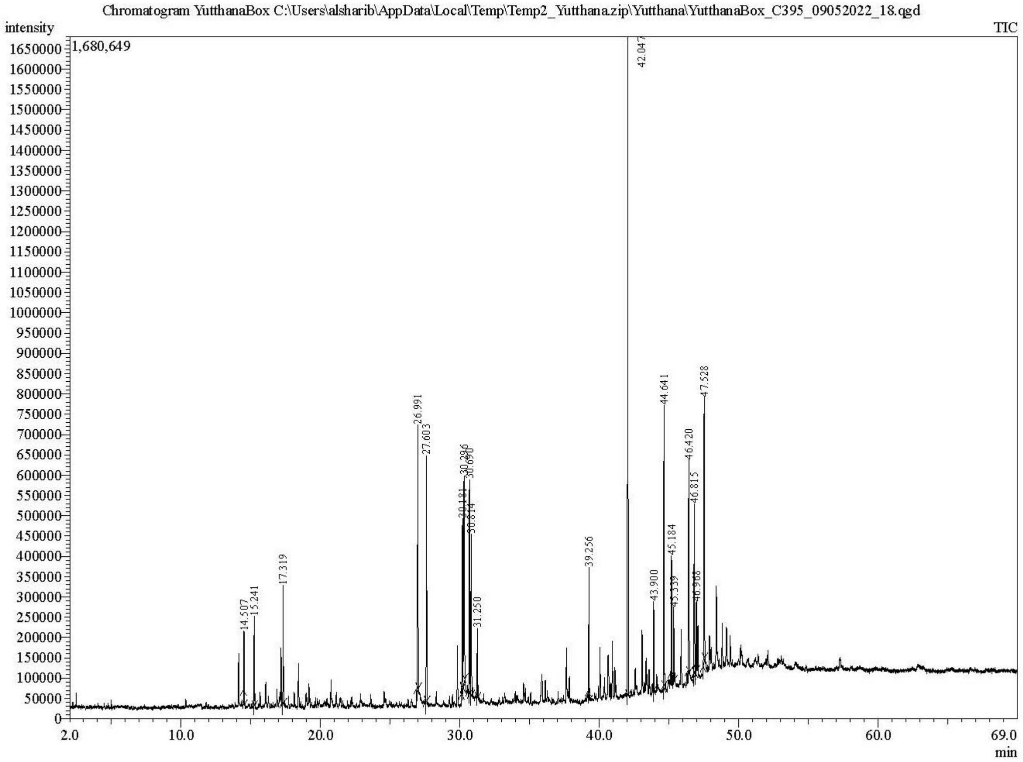

Peak Report TIC

Peak#	R.Time	I.Time	F.Time	Area	Area%	Height	Height%	Name
1	14.507	14.475	14.555	348686	1.34	159272	1.77	Cyclohexane, 1-ethenyl-1-methyl-2,4-bis(1-me
2	15.241	15.200	15.295	551387	2.11	215845	2.39	Caryophyllene
3	17.319	17.275	17.370	669000	2.56	288627	3.20	.beta.-Bisabolene
4	26.991	26.905	27.040	2167409	8.31	648560	7.19	n-Hexadecanoic acid
5	27.603	27.550	27.655	1504367	5.77	605419	6.71	Hexadecanoic acid, ethyl ester
6	30.181	30.115	30.230	1400469	5.37	422878	4.69	Linoelaidic acid
7	30.296	30.230	30.380	1911887	7.33	512802	5.69	cis,cis,cis-7,10,13-Hexadecatrienal
8	30.690	30.630	30.750	1586401	6.08	522745	5.80	9,12-Octadecadienoic acid (Z,Z)-
9	30.814	30.750	30.865	1124705	4.31	394742	4.38	9,12,15-Octadecatrienoic acid, (Z,Z,Z)-
10	31.250	31.215	31.300	373679	1.43	164017	1.82	Eicosanoic acid, ethyl ester
11	39.256	39.210	39.310	794052	3.04	314672	3.49	Tetracosane
12	42.047	41.980	42.110	4359806	16.71	1619193	17.96	Tetratetracontane
13	43.900	43.855	43.955	583517	2.24	217936	2.42	Octacosanal
14	44.641	44.585	44.700	1896329	7.27	692276	7.68	Hentriacontane
15	45.184	45.135	45.240	806268	3.09	303137	3.36	Vitamin E
16	45.339	45.300	45.390	413145	1.58	169442	1.88	
17	46.420	46.360	46.495	1821030	6.98	521766	5.79	Ergost-5-en-3-ol, (3.beta.)-
18	46.815	46.760	46.875	1249041	4.79	418263	4.64	Stigmasterol
19	46.968	46.875	47.020	429954	1.65	171197	1.90	2-Cyclohexen-3,5-diol-1-one, 2-[11-phenylunc
20	47.528	47.460	47.595	2092982	8.02	653570	7.25	.gamma.-Sitosterol
				26084114	100.00	9016359	100.00	

Figure S3 The GC/MS spectrum and reported data of *P. cavaleriei* (c395) from the library.

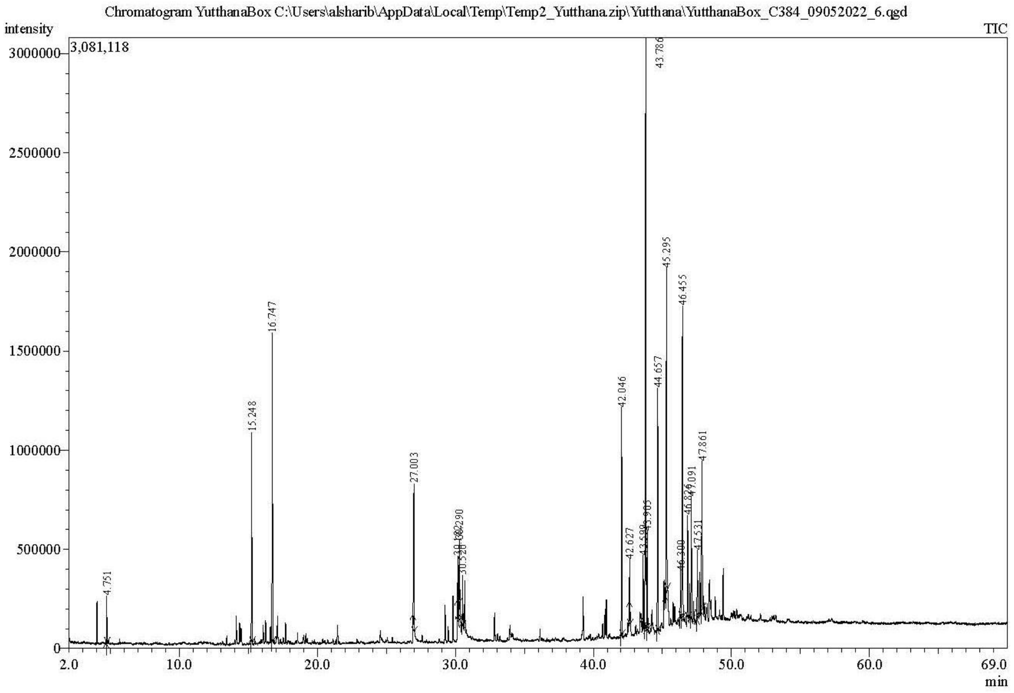

Peak Report TIC

Peak#	R.Time	I.Time	F.Time	Area	Area%	Height	Height%	Name
1	4.751	4.720	4.790	401588	0.83	236142	1.43	Bicyclo[3.1.1]heptane, 6,6-dimethyl-2-methyl
2	15.248	15.195	15.305	2726786	5.66	1051198	6.38	Caryophyllene
3	16.747	16.690	16.805	3965916	8.24	1548603	9.40	Germacrene D
4	27.003	26.930	27.045	2210800	4.59	714556	4.34	n-Hexadecanoic acid
5	30.182	30.150	30.230	621480	1.29	263687	1.60	Linoelaidic acid
6	30.290	30.245	30.335	914508	1.90	340647	2.07	7-Tetradecenal, (Z)-
7	30.520	30.485	30.565	558953	1.16	245089	1.49	trans-13-Octadecenoic acid
8	42.046	41.990	42.105	2888278	6.00	1142978	6.94	Tetratetracontane
9	42.627	42.595	42.680	571271	1.19	255433	1.55	2-Cyclohexen-3-ol-1-one, 2-[11-tetradecenoyl
10	43.599	43.560	43.650	783678	1.63	336320	2.04	2-Cyclohexen-3,6-diol-1-one, 2-[10-tetradecen
11	43.786	43.700	43.860	10064053	20.90	2977610	18.08	2-Cyclohexen-3,6-diol-1-one, 2-[10-tetradecen
12	43.905	43.860	43.960	1230271	2.56	494989	3.01	Octacosanal
13	44.657	44.595	44.715	3414409	7.09	1218201	7.40	Hentriacontane
14	45.295	45.225	45.385	5078781	10.55	1637256	9.94	1-nonanone, 1-(2,5-dihydroxyphenyl)-
15	46.300	46.275	46.390	673427	1.40	227192	1.38	2-Cyclohexen-3,6-diol-1-one, 2-[10-tetradecen
16	46.455	46.390	46.520	5192784	10.79	1568116	9.52	2-Cyclohexen-3,6-diol-1-one, 2-[10-tetradecen
17	46.826	46.780	46.890	1494142	3.10	506139	3.07	Stigmasterol
18	47.091	47.040	47.155	1764470	3.66	615818	3.74	Hentriacontane
19	47.531	47.485	47.590	1004993	2.09	339135	2.06	.gamma.-Sitosterol
20	47.861	47.800	47.955	2585553	5.37	752243	4.57	1-nonanone, 1-(2,5-dihydroxyphenyl)-
				48146141	100.00	16471352	100.00	

Figure S4 The GC/MS spectrum and reported data of *P. dindygulensis (*c384) from the library.

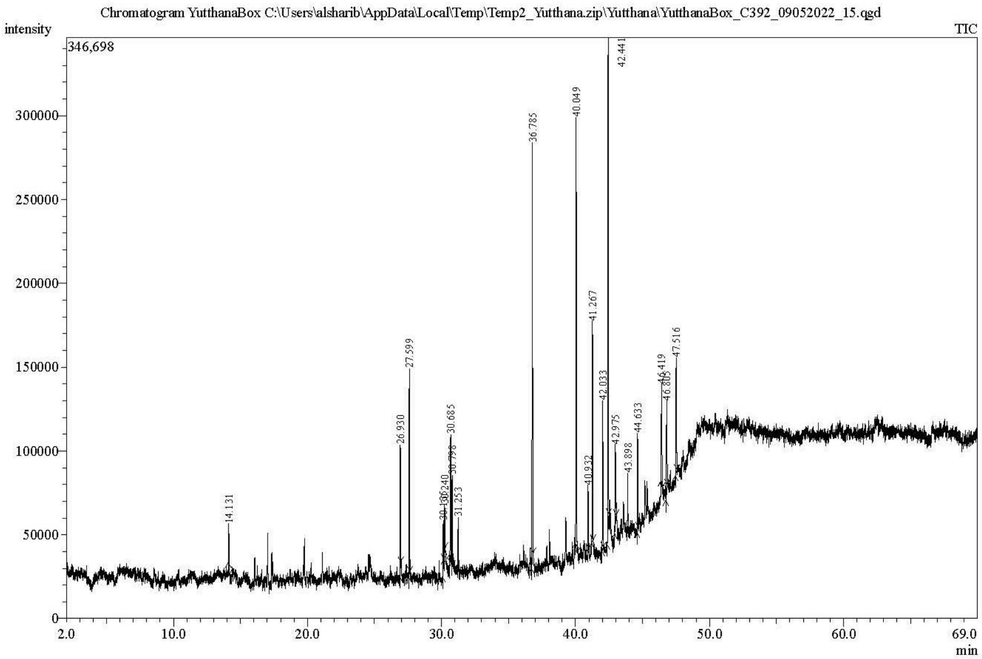

Peak Report TIC

Peak#	R.Time	I.Time	F.Time	Area	Area%	Height	Height%	Name
1	14.131	14.100	14.170	53028	1.02	26446	1.45	Copaene
2	26.930	26.880	26.985	202044	3.87	74175	4.08	n-Hexadecanoic acid
3	27.599	27.545	27.650	292003	5.60	122449	6.73	Hexadecanoic acid, ethyl ester
4	30.135	30.090	30.205	85439	1.64	29274	1.61	Linoelaidic acid
5	30.240	30.205	30.280	73955	1.42	29303	1.61	7-Tetradecenal, (Z)-
6	30.685	30.625	30.735	219094	4.20	74194	4.08	Linoleic acid ethyl ester
7	30.798	30.755	30.855	156183	2.99	53101	2.92	9,12,15-Octadecatrienoic acid, 2,3-dihydroxyp
8	31.253	31.220	31.295	67445	1.29	30595	1.68	Octadecanoic acid, ethyl ester
9	36.785	36.715	36.845	747619	14.33	248737	13.67	2-Cyclohexen-3-ol-1-one, 2-[11-phenylundeca
10	40.049	39.995	40.130	710934	13.63	256424	14.10	2-Cyclohexen-3-ol-1-one, 2-[9-phennonanoyl]
11	40.932	40.895	40.985	95855	1.84	38373	2.11	2-Cyclohexen-3,6-diol-1-one, 2-tetradecanoyl-
12	41.267	41.210	41.320	363988	6.98	131429	7.23	
13	42.033	41.985	42.090	248950	4.77	88675	4.87	Tetratetracontane
14	42.441	42.375	42.495	908800	17.43	292244	16.07	
15	42.975	42.920	43.050	176789	3.39	48523	2.67	Phen-1,5-diol, 2-[9-phennonanoyl]-
16	43.898	43.860	43.950	76421	1.47	33561	1.85	Octacosanal
17	44.633	44.590	44.685	149234	2.86	57759	3.18	Tetratetracontane
18	46.419	46.370	46.485	187666	3.60	59682	3.28	Campesterol
19	46.805	46.750	46.870	182954	3.51	55026	3.03	Stigmasterol
20	47.516	47.470	47.590	217010	4.16	69020	3.79	.gamma.-Sitosterol
				5215411	100.00	1818990	100.00	

Figure S5 The GC/MS spectrum and reported data of *P. heptaphylla (*c392) from the library.

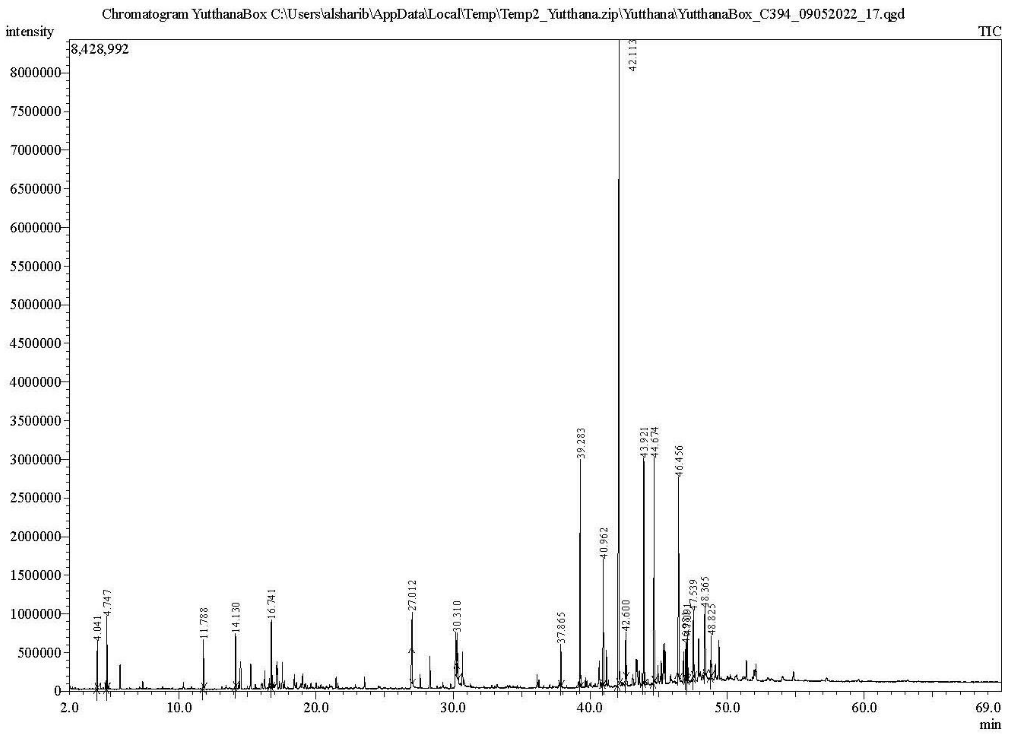

Peak Report TIC

Peak#	R.Time	I.Time	F.Time	Area	Area%	Height	Height%	Name
1	4.041	4.010	4.080	940622	1.02	616961	2.02	.alpha.-Pinene
2	4.747	4.710	4.790	1631661	1.77	937045	3.07	.beta.-Pinene
3	11.788	11.745	11.840	1478155	1.61	627382	2.06	Acetic acid, 1,7,7-trimethyl-bicyclo[2.2.1]hept
4	14.130	14.085	14.185	1651063	1.79	684396	2.24	Copaene
5	16.741	16.695	16.795	2087008	2.27	856694	2.81	(1R,2S,6S,7S,8S)-8-Isopropyl-1-methyl-3-met
6	27.012	26.970	27.055	1312082	1.43	690011	2.26	n-Hexadecanoic acid
7	30.310	30.270	30.385	1180530	1.28	469142	1.54	7-Tetradecenal, (Z)-
8	37.865	37.820	37.910	1252195	1.36	523571	1.72	2-Cyclohexen-3,6-diol-1-one, 2-tetradecanoyl-
9	39.283	39.210	39.335	8241045	8.96	2917050	9.56	Tetracosane
10	40.962	40.900	41.015	4379608	4.76	1612467	5.28	2-Cyclohexen-3,6-diol-1-one, 2-tetradecanoyl-
11	42.113	41.985	42.160	31911168	34.69	8337654	27.32	Tetratetracontane
12	42.600	42.550	42.650	1766338	1.92	610464	2.00	1-nonanone, 1-(2,5-dihydroxyphenyl)-
13	43.921	43.855	43.980	7941339	8.63	2911131	9.54	Octacosanal
14	44.674	44.595	44.725	8566111	9.31	2894479	9.48	Hentriacontane
15	46.456	46.385	46.515	7765569	8.44	2574549	8.44	Octacosanal
16	46.981	46.935	47.030	1155588	1.26	457313	1.50	
17	47.091	47.030	47.145	1537506	1.67	578514	1.90	Hentriacontane
18	47.539	47.485	47.600	2518031	2.74	853445	2.80	.gamma.-Sitosterol
19	48.365	48.310	48.470	3242414	3.53	839529	2.75	
20	48.825	48.780	48.880	1423473	1.55	529696	1.74	Dotriacontanal
				91981506	100.00	30521493	100.00	

Figure S6 The GC/MS spectrum and reported data of *P. heyeana* (c394) from the library.

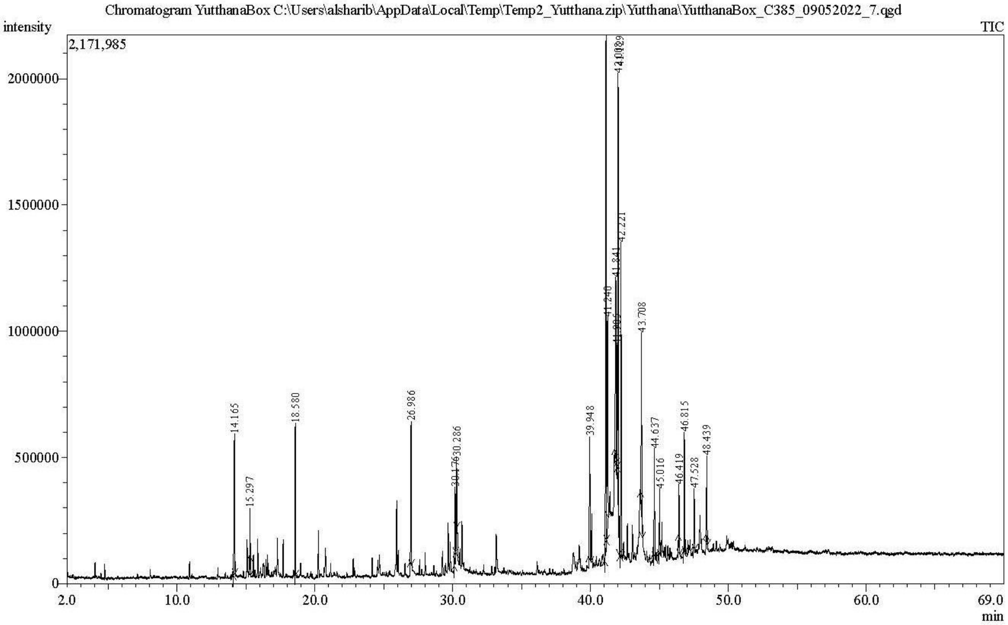

Peak Report TIC

Peak#	R.Time	I.Time	F.Time	Area	Area%	Height	Height%	Name
1	14.165	14.085	14.225	2239397	5.62	555727	4.38	Geranyl acetate
2	15.297	15.265	15.350	470944	1.18	217625	1.72	1H-3a,7-Methanoazulene, octahydro-3,8,8-trin
3	18.580	18.530	18.630	1363730	3.42	595772	4.70	1,6,10-Dodecatrien-3-ol, 3,7,11-trimethyl-, (E)
4	26.986	26.915	27.035	1761878	4.42	563851	4.45	n-Hexadecanoic acid
5	30.176	30.120	30.225	919018	2.30	283979	2.24	Linoelaidic acid
6	30.286	30.230	30.330	895800	2.25	322646	2.54	7-Tetradecenal, (Z)-
7	39.948	39.880	40.025	1861790	4.67	484813	3.82	A-Norcholestan-2-one, (5.alpha.)-
8	41.129	41.045	41.170	6224345	15.61	2026219	15.98	7-Oxabicyclo[4.1.0]heptane, 1-(2,3-dimethyl-1
9	41.240	41.170	41.310	3502045	8.78	838056	6.61	Epicholestanol
10	41.841	41.765	41.900	3687092	9.25	712316	5.62	
11	41.905	41.900	41.940	471365	1.18	471466	3.72	3-Hexen-1-ol, 6-(2,6,6-trimethyl-1-cyclohexen
12	42.008	41.940	42.095	5734529	14.38	1712185	13.50	2-Heptanone, 6-(3,5-dimethyl-2-furanyl)-6-me
13	42.221	42.155	42.280	3210505	8.05	1243643	9.81	Triphenylene, octadecahydro-
14	43.708	43.645	43.795	2070314	5.19	706016	5.57	Chrom-5-ol-4-one, 2-decyl-
15	44.637	44.585	44.695	1222215	3.07	422058	3.33	Tetracontane, 3,5,24-trimethyl-
16	45.016	44.980	45.070	613479	1.54	255966	2.02	Naphthalene, 1,1'-(1,10-decanediyl)bis[decahy
17	46.419	46.380	46.485	679838	1.71	229173	1.81	Ergost-5-en-3-ol, (3.beta.)-
18	46.815	46.765	46.880	1419589	3.56	472651	3.73	Stigmasterol
19	47.528	47.480	47.580	661322	1.66	234920	1.85	.gamma.-Sitosterol
20	48.439	48.395	48.500	861996	2.16	329846	2.60	1-Adamantanecarboxylic acid, 2-(1-adamantyl
				39871191	100.00	12678928	100.00	

Figure S7 The GC/MS spectrum and reported data of *P. kotana* (c385) from the library.

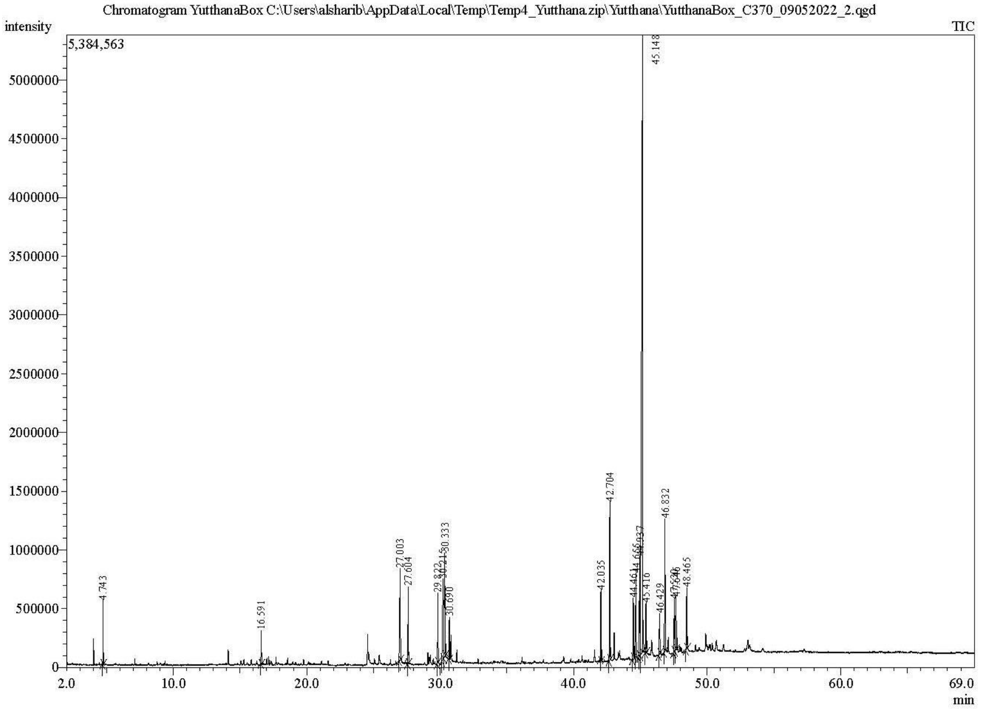

Peak Report TIC

Peak#	R.Time	I.Time	F.Time	Area	Area%	Height	Height%	Name
1	4.743	4.705	4.790	935931	1.43	544375	3.12	Bicyclo[3.1.1]heptane, 6,6-dimethyl-2-methyl(
2	16.591	16.545	16.645	693619	1.06	284978	1.63	.gamma.-Muurolene
3	27.003	26.900	27.065	3031507	4.64	778170	4.46	n-Hexadecanoic acid
4	27.604	27.545	27.660	1705978	2.61	655003	3.76	Hexadecanoic acid, ethyl ester
5	29.822	29.770	29.885	1488282	2.28	596860	3.42	Phytol
6	30.215	30.110	30.260	3313719	5.07	682248	3.91	Linoelaidic acid
7	30.333	30.260	30.430	3598660	5.51	890101	5.11	7-Tetradecenal, (Z)-
8	30.690	30.635	30.750	1011084	1.55	359583	2.06	9,12-Octadecadienoic acid (Z,Z)-
9	42.035	41.985	42.100	1583962	2.43	595923	3.42	Tetratetracontane
10	42.704	42.630	42.770	3647386	5.58	1347001	7.73	Phen-1,3-diol, 2-dodecanoyl-
11	44.461	44.395	44.525	1445936	2.21	518733	2.98	Chrom-5-ol-4-one, 2-undecyl-
12	44.655	44.595	44.730	2081819	3.19	720678	4.13	Hentriacontane
13	44.937	44.860	44.975	2590323	3.97	852217	4.89	Chrom-7-ol-4-one, 2-nonyl-
14	45.148	44.975	45.180	27201766	41.65	5259164	30.17	Phen-1,3-diol, 2-dodecanoyl-
15	45.416	45.350	45.500	1232741	1.89	425518	2.44	Phen-1,3-diol, 2-dodecanoyl-
16	46.429	46.355	46.505	1200449	1.84	349714	2.01	Campesterol
17	46.832	46.755	46.905	3592163	5.50	1131295	6.49	6S-2,3,8,8-Tetramethyltricyclo[5.2.2.0(1,6)]un
18	47.530	47.460	47.585	1408903	2.16	454023	2.60	.gamma.-Sitosterol
19	47.646	47.585	47.750	1990801	3.05	460431	2.64	Phen-1,3-diol, 2-dodecanoyl-
20	48.465	48.395	48.540	1559221	2.39	525580	3.02	Phen-1,5-diol, 2-[9-phennonanoyl]-
				65314250	100.00	17431595	100.00	

Figure S8 The GC/MS spectrum and reported data of *P. laevifolia* (c370) from the library.

Chromatogram YutthanaBox C:\Users\alsharib\AppData\Local\Temp\Temp2_Yutthana.zip\Yutthana\YutthanaBox_C371_09052022_3.qgd

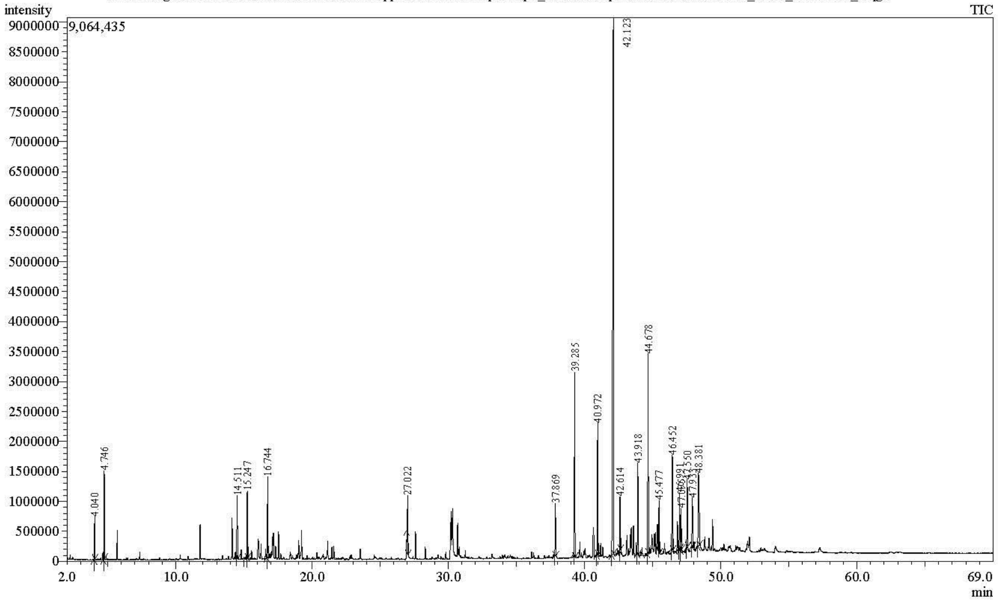

Peak Report TIC

Peak#	R.Time	I.Time	F.Time	Area	Area%	Height	Height%	Name
1	4.040	4.010	4.080	1102354	1.02	713052	2.07	.alpha.-Pinene
2	4.746	4.705	4.790	2535048	2.35	1473879	4.28	Bicyclo[3.1.1]heptane, 6,6-dimethyl-2-methyl(
3	14.511	14.440	14.560	3207935	2.97	1021723	2.97	Cyclohexane, 1-ethenyl-1-methyl-2,4-bis(1-me
4	15.247	15.195	15.300	2826416	2.62	1101760	3.20	Caryophyllene
5	16.744	16.690	16.800	3453666	3.20	1346963	3.91	(1R,2S,6S,7S,8S)-8-Isopropyl-1-methyl-3-met
6	27.022	26.965	27.060	1934336	1.79	824679	2.40	n-Hexadecanoic acid
7	37.869	37.820	37.920	2249725	2.08	858608	2.49	2-Cyclohexen-3,6-diol-1-one, 2-tetradecanoyl-
8	39.285	39.215	39.335	8809992	8.16	3062232	8.89	Tetracosane
9	40.972	40.900	41.025	6367272	5.90	2195081	6.37	2-Cyclohexen-3,6-diol-1-one, 2-tetradecanoyl-
10	42.123	41.990	42.170	35637716	33.01	8943734	25.97	Tetratetracontane
11	42.614	42.560	42.670	2587720	2.40	888596	2.58	1-nonanone, 1-(2,5-dihydroxyphenyl)-
12	43.918	43.860	43.970	4045371	3.75	1517249	4.41	Octacosanal
13	44.678	44.600	44.730	9601560	8.89	3326369	9.66	Tetratetracontane
14	45.477	45.420	45.525	2249801	2.08	824052	2.39	(+)-Sesamin
15	46.452	46.380	46.515	5474743	5.07	1578967	4.59	E,E-2,13-Octadecadien-1-ol
16	46.991	46.940	47.040	2372452	2.20	934691	2.71	
17	47.095	47.040	47.150	1817575	1.68	686701	1.99	Hentriacontane
18	47.550	47.490	47.605	3241698	3.00	1122976	3.26	.gamma.-Sitosterol
19	47.933	47.870	47.990	2605338	2.41	812390	2.36	
20	48.381	48.315	48.485	5838641	5.41	1199313	3.48	
				107959359	100.00	34433015	100.00	

Figure S9 The GC/MS spectrum and reported data of *P. masuthoniana (*c371) from the library.

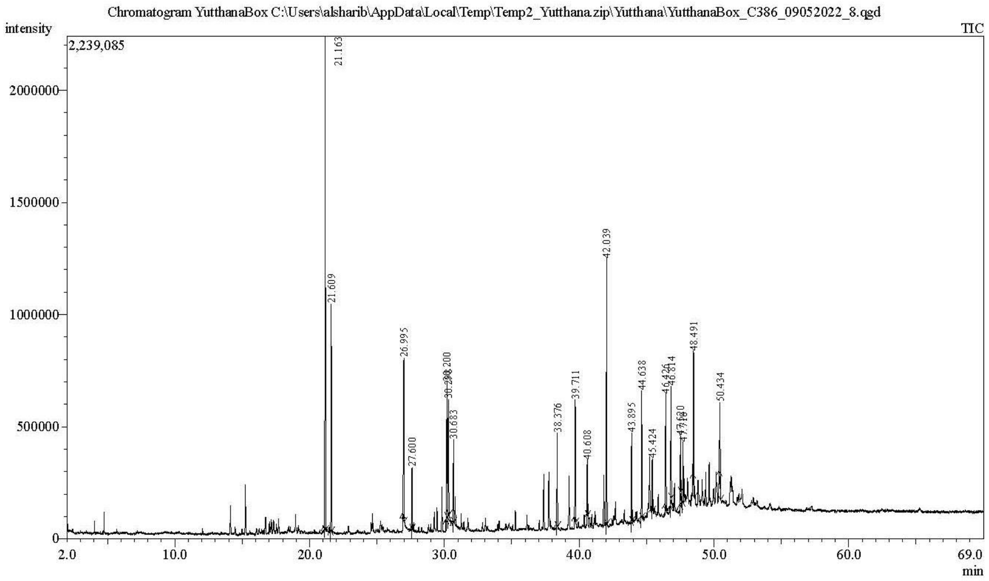

Peak Report TIC

Peak#	R.Time	I.Time	F.Time	Area	Area%	Height	Height%	Name
1	21.163	21.085	21.215	5687423	17.30	2193555	18.67	8-Heptadecene
2	21.609	21.555	21.665	2483871	7.55	1011561	8.61	Eicosane
3	26.995	26.915	27.045	2419688	7.36	716353	6.10	n-Hexadecanoic acid
4	27.600	27.560	27.650	635331	1.93	268914	2.29	Hexadecanoic acid, ethyl ester
5	30.200	30.120	30.240	2269531	6.90	606099	5.16	Linoelaidic acid
6	30.298	30.240	30.385	1918910	5.84	521079	4.44	7-Tetradecenal, (Z)-
7	30.683	30.635	30.740	1048647	3.19	363183	3.09	9,12-Octadecadienoic acid (Z,Z)-
8	38.376	38.325	38.425	1112551	3.38	411886	3.51	(E)-1-(2-Hydroxy-4,6-dimethoxyphenyl)-3-ph
9	39.711	39.655	39.760	1493467	4.54	535283	4.56	Flavone, 5-hydroxy-7,8-dimethoxy-
10	40.608	40.560	40.655	706970	2.15	244545	2.08	4H-1-Benzopyran-4-one, 5,7-dimethoxy-2-phe
11	42.039	41.975	42.095	3068444	9.33	1180133	10.05	Tetratetracontane
12	43.895	43.850	43.950	971654	2.96	392632	3.34	Octacosanal
13	44.638	44.590	44.700	1520787	4.63	562774	4.79	Hentriacontane
14	45.424	45.385	45.480	573888	1.75	223509	1.90	2-Cyclohexen-3-ol-1-one, 2-dodecanoyl-
15	46.426	46.365	46.490	1675446	5.10	506642	4.31	Octacosanal
16	46.814	46.760	46.870	1421555	4.32	517590	4.41	Stigmasterol
17	47.520	47.475	47.570	703753	2.14	280572	2.39	.gamma.-Sitosterol
18	47.710	47.665	47.760	677198	2.06	267290	2.28	2-Cyclohexen-3-ol-1-one, 2-[11-tetradecenoyl]
19	48.491	48.450	48.550	1336901	4.07	589638	5.02	2-Cyclohexen-3-ol-1-one, 2-[11-phenylundeca
20	50.434	50.390	50.510	1152674	3.51	352880	3.00	
				32878689	100.00	11746118	100.00	

Figure S10 The GC/MS spectrum and reported data of *P. moulmeiniana* (c386) from the library.

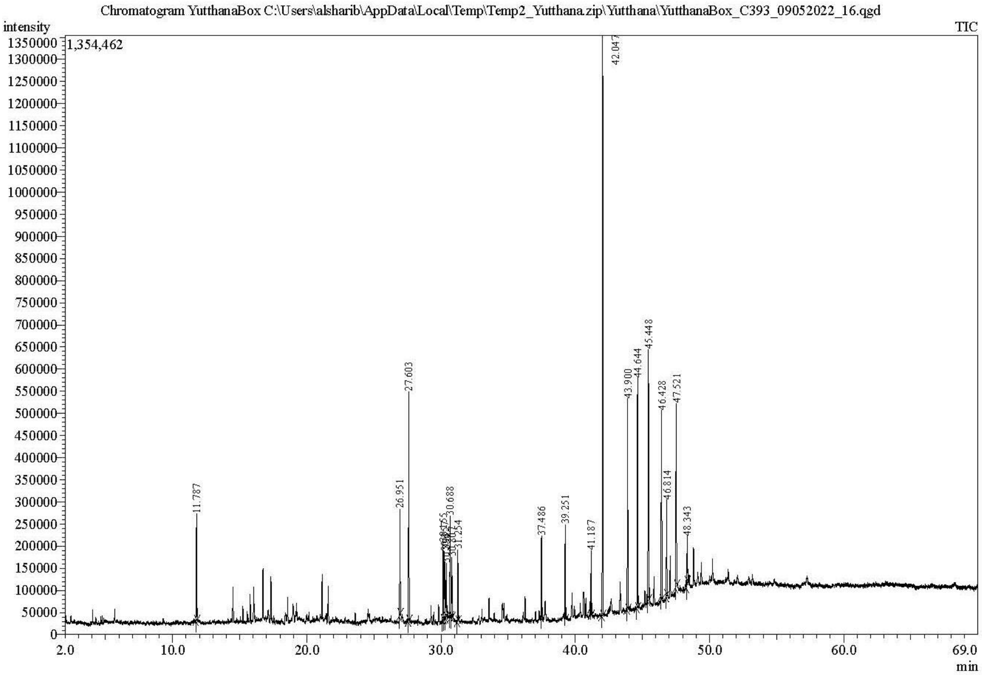

Peak Report TIC

Peak#	R.Time	I.Time	F.Time	Area	Area%	Height	Height%	Name
1	11.787	11.740	11.845	615651	3.30	241021	3.69	Acetic acid, 1,7,7-trimethyl-bicyclo[2.2.1]hept
2	26.951	26.890	27.005	615285	3.30	238654	3.65	n-Hexadecanoic acid
3	27.603	27.540	27.660	1346079	7.22	517137	7.92	Hexadecanoic acid, ethyl ester
4	30.155	30.095	30.205	520402	2.79	173526	2.66	Linoelaidic acid
5	30.257	30.205	30.320	584494	3.14	150859	2.31	7-Tetradecenal, (Z)-
6	30.396	30.320	30.455	380423	2.04	122210	1.87	(2E,4E)-N-Isobutyldodeca-2,4-dienamide
7	30.688	30.610	30.745	722920	3.88	226369	3.47	Linoelaidic acid
8	30.807	30.745	30.865	408216	2.19	134912	2.07	cis,cis,cis-7,10,13-Hexadecatrienal
9	31.254	31.205	31.305	391007	2.10	161396	2.47	Octadecanoic acid, ethyl ester
10	37.486	37.435	37.545	497272	2.67	185270	2.84	
11	39.251	39.200	39.315	548072	2.94	207311	3.17	Tetracosane
12	41.187	41.145	41.240	337802	1.81	137154	2.10	Hexacosanal
13	42.047	41.965	42.105	3538299	18.98	1310814	20.07	Tetratetracontane
14	43.900	43.840	43.970	1280905	6.87	475459	7.28	Octacosanal
15	44.644	44.580	44.705	1426123	7.65	519043	7.95	Hentriacontane
16	45.448	45.380	45.520	1795180	9.63	566631	8.67	(+)-Sesamin
17	46.428	46.355	46.500	1448821	7.77	424739	6.50	Octacosanal
18	46.814	46.755	46.875	588670	3.16	216824	3.32	Stigmasterol
19	47.521	47.460	47.600	1266933	6.80	414298	6.34	.gamma.-Sitosterol
20	48.343	48.290	48.395	330192	1.77	109201	1.67	
				18642746	100.00	6532828	100.00	

Figure S11 The GC/MS spectrum and reported data of *P. multisurcula (*c393) from the library.

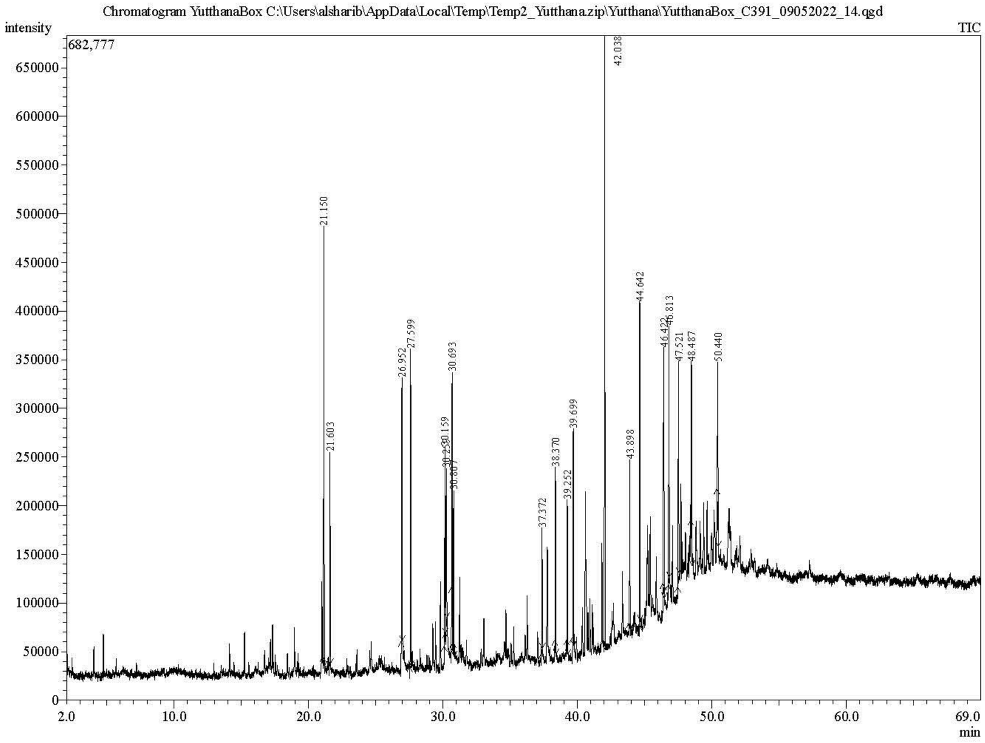

Peak Report TIC

Peak#	R.Time	I.Time	F.Time	Area	Area%	Height	Height%	Name
1	21.150	21.090	21.205	1166721	8.77	447289	9.11	8-Heptadecene
2	21.603	21.560	21.650	515376	3.88	217632	4.43	Eicosane
3	26.952	26.900	27.000	682435	5.13	270654	5.51	n-Hexadecanoic acid
4	27.599	27.550	27.655	826879	6.22	325750	6.63	Hexadecanoic acid, ethyl ester
5	30.159	30.110	30.205	523129	3.93	198062	4.03	Linoelaidic acid
6	30.259	30.205	30.315	486557	3.66	161305	3.28	7-Tetradecenal, (Z)-
7	30.693	30.645	30.745	618791	4.65	252174	5.13	Linoleic acid ethyl ester
8	30.807	30.760	30.860	462577	3.48	162665	3.31	cis,cis,cis-7,10,13-Hexadecatrienal
9	37.372	37.325	37.420	354263	2.66	126679	2.58	4H-1-Benzopyran-4-one, 2,3-dihydro-5,7-dim
10	38.370	38.325	38.425	504348	3.79	184281	3.75	(E)-1-(2-Hydroxy-4,6-dimethoxyphenyl)-3-ph
11	39.252	39.210	39.305	381819	2.87	150478	3.06	Tetracosane
12	39.699	39.650	39.755	600124	4.51	218215	4.44	Flavone, 5-hydroxy-7,8-dimethoxy-
13	42.038	41.980	42.100	1663945	12.51	625779	12.74	Tetratetracontane
14	43.898	43.860	43.950	419612	3.15	170238	3.47	Octacosanal
15	44.642	44.590	44.695	850252	6.39	329577	6.71	Hentriacontane
16	46.422	46.370	46.490	886748	6.67	248721	5.06	Ergost-5-en-3-ol, (3.beta.)-
17	46.813	46.765	46.875	767052	5.77	262906	5.35	Stigmasterol
18	47.521	47.470	47.590	671715	5.05	225373	4.59	.gamma.-Sitosterol
19	48.487	48.450	48.550	484329	3.64	179916	3.66	2-Cyclohexen-3-ol-1-one, 2-[11-phenylundeca
20	50.440	50.395	50.505	433280	3.26	154231	3.14	2,6-dimethoxy-4-(4-(3,4,5-trimethoxyphenyl)t
				13299952	100.00	4911925	100.00	

Figure S12 The GC/MS spectrum and reported data of *P. nakaharai (*c391) from the library.

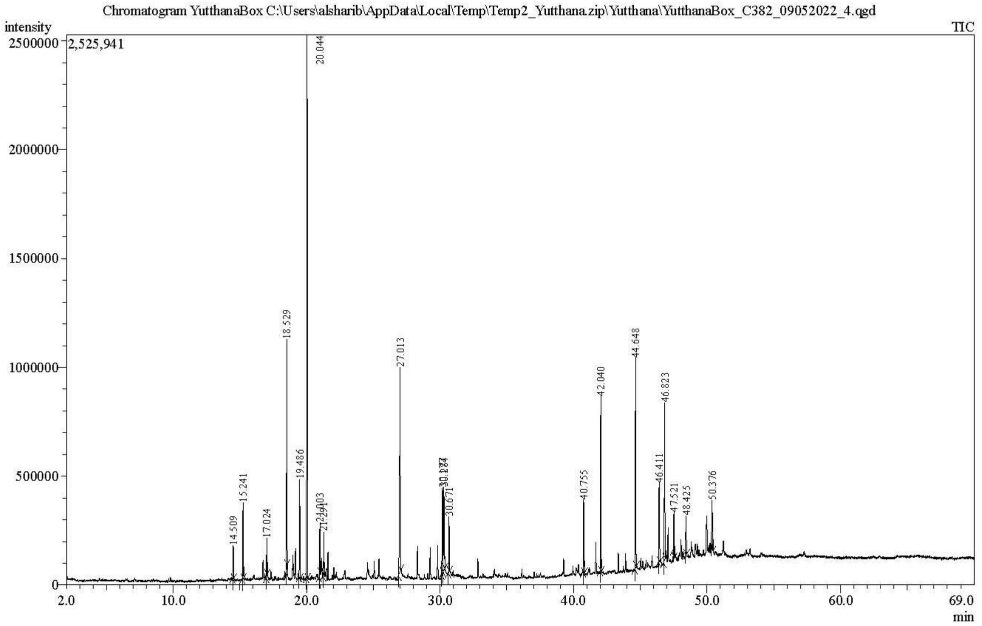

Peak Report TIC

Peak#	R.Time	I.Time	F.Time	Area	Area%	Height	Height%	Name
1	14.509	14.465	14.555	362527	1.15	150553	1.39	Cyclohexane, 1-ethenyl-1-methyl-2,4-bis(1-me
2	15.241	15.190	15.300	908272	2.87	348406	3.22	Caryophyllene
3	17.024	16.985	17.070	410207	1.30	172928	1.60	Pentadecane
4	18.529	18.465	18.570	2483989	7.86	1046951	9.66	1,2-Dimethoxy-4-(2-methoxyethenyl)benzene
5	19.486	19.430	19.540	1185673	3.75	451694	4.17	Carotol
6	20.044	19.960	20.100	7147709	22.61	2494029	23.02	Apiol
7	21.003	20.955	21.050	600010	1.90	238602	2.20	2-Cyclohexen-3-ol-1-one, 2-dodecanoyl-
8	21.291	21.250	21.345	464076	1.47	199485	1.84	Apiol
9	27.013	26.895	27.075	3645395	11.53	939203	8.67	n-Hexadecanoic acid
10	30.177	30.110	30.225	1342702	4.25	390570	3.60	Linoelaidic acid
11	30.284	30.225	30.385	1373798	4.35	382099	3.53	7-Tetradecenal, (Z)-
12	30.671	30.625	30.735	667235	2.11	254997	2.35	Octadecanoic acid
13	40.755	40.705	40.840	954842	3.02	334533	3.09	
14	42.040	41.980	42.100	2151194	6.81	803127	7.41	Tetratetracontane
15	44.648	44.585	44.715	2583873	8.17	963525	8.89	Hentriacontane
16	46.411	46.355	46.490	1268775	4.01	363399	3.35	Ergost-5-en-3-ol, (3.beta.)-
17	46.823	46.755	46.895	2225867	7.04	725166	6.69	Stigmasterol
18	47.521	47.475	47.580	538177	1.70	189173	1.75	.gamma.-Sitosterol
19	48.425	48.375	48.485	545779	1.73	171542	1.58	4,22-Stigmastadiene-3-one
20	50.376	50.320	50.450	747656	2.37	215382	1.99	
				31607756	100.00	10835364	100.00	

Figure S13 The GC/MS spectrum and reported data of *P. pellucida (*c382) from the library.

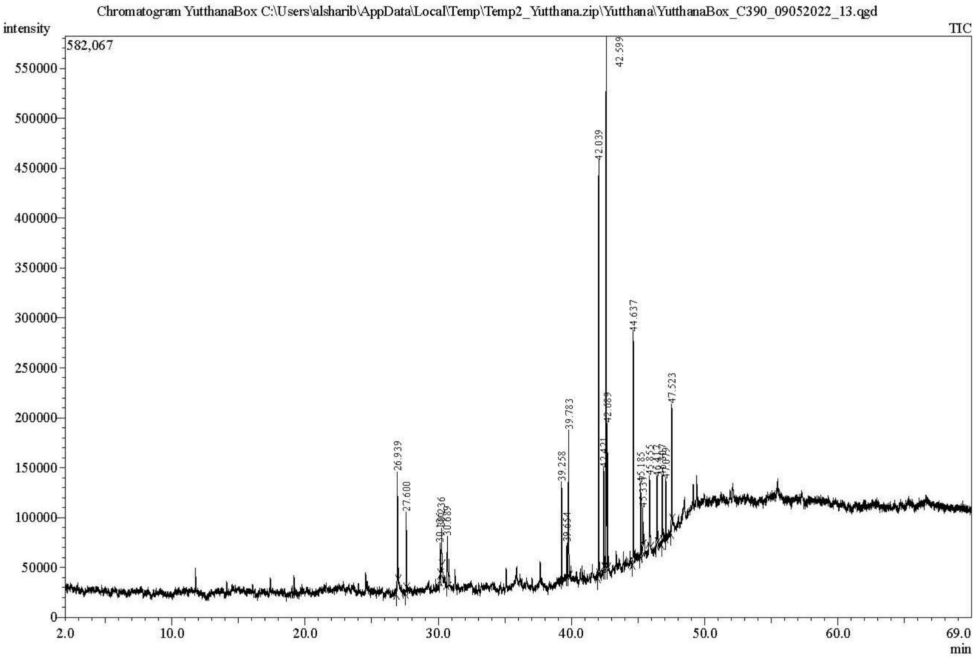

Peak Report TIC

Peak#	R.Time	I.Time	F.Time	Area	Area%	Height	Height%	Name
1	26.939	26.870	27.005	325427	4.52	115437	4.57	n-Hexadecanoic acid
2	27.600	27.550	27.645	189351	2.63	78471	3.11	Hexadecanoic acid, ethyl ester
3	30.136	30.100	30.185	89041	1.24	34867	1.38	Linoelaidic acid
4	30.236	30.185	30.295	126953	1.76	42009	1.66	cis-7-Hexadecenoic acid
5	30.689	30.610	30.745	193794	2.69	49969	1.98	Linoelaidic acid
6	39.258	39.210	39.310	248513	3.45	98298	3.89	Tetratetracontane
7	39.654	39.610	39.710	92569	1.29	33513	1.33	Phen-1,3-diol, 2-dodecanoyl-
8	39.783	39.725	39.860	394615	5.48	145553	5.76	Phen-1,3-diol, 2-tetradecanoyl-
9	42.039	41.975	42.105	1098996	15.27	416027	16.48	Tetratetracontane
10	42.421	42.375	42.485	244217	3.39	99936	3.96	Phen-1,3-diol, 2-dodecanoyl-
11	42.599	42.485	42.650	1570728	21.82	532167	21.08	Phen-1,3-diol, 2-dodecanoyl-
12	42.689	42.650	42.760	423038	5.88	144724	5.73	Phen-1,3-diol, 2-dodecanoyl-
13	44.637	44.575	44.710	633233	8.80	232337	9.20	Hentriacontane
14	45.185	45.135	45.255	211814	2.94	74675	2.96	Phen-1,3-diol, 2-dodecanoyl-
15	45.337	45.295	45.395	116982	1.63	41205	1.63	Phen-1,3-diol, 2-dodecanoyl-
16	45.855	45.800	45.940	246584	3.43	69609	2.76	Phen-1,3-diol, 2-[11-phenundecanoyl]-
17	46.417	46.355	46.480	236822	3.29	67554	2.68	Ergost-5-en-3-ol, (3.beta.)-
18	46.807	46.760	46.870	202807	2.82	67152	2.66	Stigmasterol
19	47.079	47.035	47.125	144566	2.01	59252	2.35	Tetratetracontane
20	47.523	47.460	47.580	406905	5.65	122299	4.84	.gamma.-Sitosterol
				7196955	100.00	2525054	100.00	

Figure S14 The GC/MS spectrum and reported data of *P. sirindhorniana (*c390) from the library.

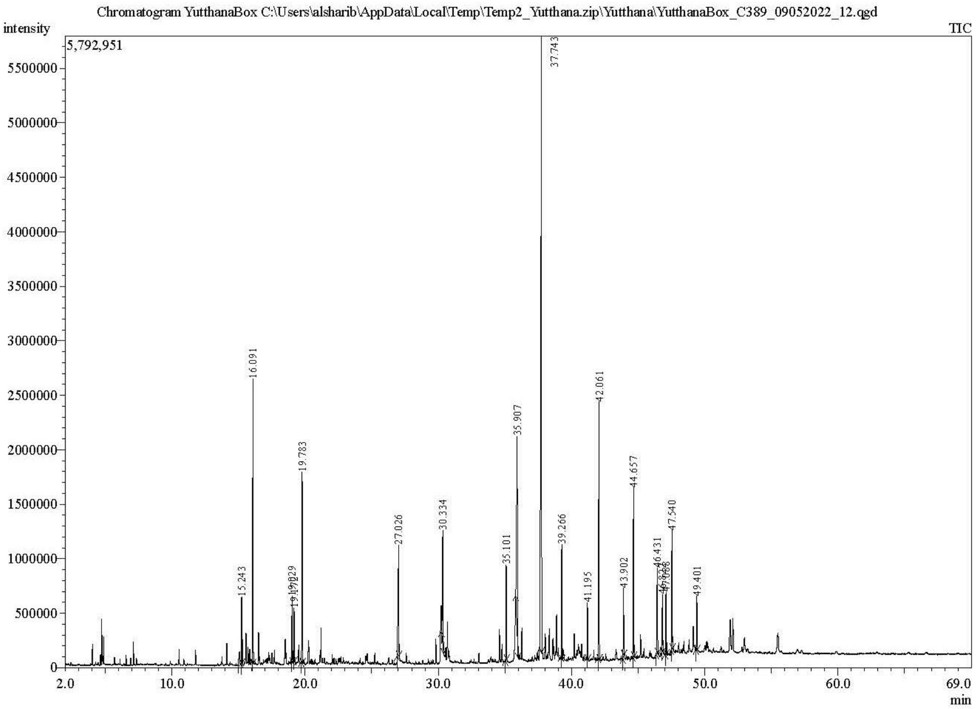

Peak Report TIC

Peak#	R.Time	I.Time	F.Time	Area	Area%	Height	Height%	Name
1	15.243	15.195	15.280	1649499	1.92	607479	2.35	Caryophyllene
2	16.091	16.025	16.145	6896689	8.03	2594320	10.03	Humulene
3	19.029	18.980	19.080	1492409	1.74	595095	2.30	(-)-Spathulenol
4	19.172	19.130	19.225	1171789	1.36	475810	1.84	Caryophyllene oxide
5	19.783	19.720	19.840	4754119	5.53	1744282	6.74	(1R,3E,7E,11R)-1,5,5,8-Tetramethyl-12-oxabi
6	27.026	26.915	27.070	4246371	4.94	1016936	3.93	n-Hexadecanoic acid
7	30.334	30.255	30.420	3619097	4.21	1023391	3.96	7-Tetradecenal, (Z)-
8	35.101	35.045	35.155	2337173	2.72	872183	3.37	
9	35.907	35.815	35.990	8119396	9.45	1758789	6.80	
10	37.743	37.590	37.785	24533636	28.56	5643342	21.82	1-(2,5-Dimethoxy-phenyl)-7-methoxy-3,11-di
11	39.266	39.210	39.320	2704500	3.15	1042211	4.03	Tetracosane
12	41.195	41.150	41.250	1321349	1.54	508178	1.96	Octacosanal
13	42.061	41.985	42.115	6635540	7.72	2357510	9.11	Tetratetracontane
14	43.902	43.850	43.955	1674583	1.95	631510	2.44	Octacosanal
15	44.657	44.590	44.710	4313557	5.02	1551636	6.00	Hentriacontane
16	46.431	46.360	46.495	2689019	3.13	785350	3.04	Ergost-5-en-3-ol, (3.beta.)-
17	46.822	46.770	46.880	1501688	1.75	513490	1.99	Stigmasterol
18	47.088	47.035	47.145	1579764	1.84	568891	2.20	Hentriacontane
19	47.540	47.475	47.600	3276554	3.81	1086599	4.20	.gamma.-Sitosterol
20	49.401	49.350	49.460	1391908	1.62	490523	1.90	Hentriacontane
				85908640	100.00	25867525	100.00	

Figure S15 The GC/MS spectrum and reported data of *P. tetraphylla (*c389) from the library.

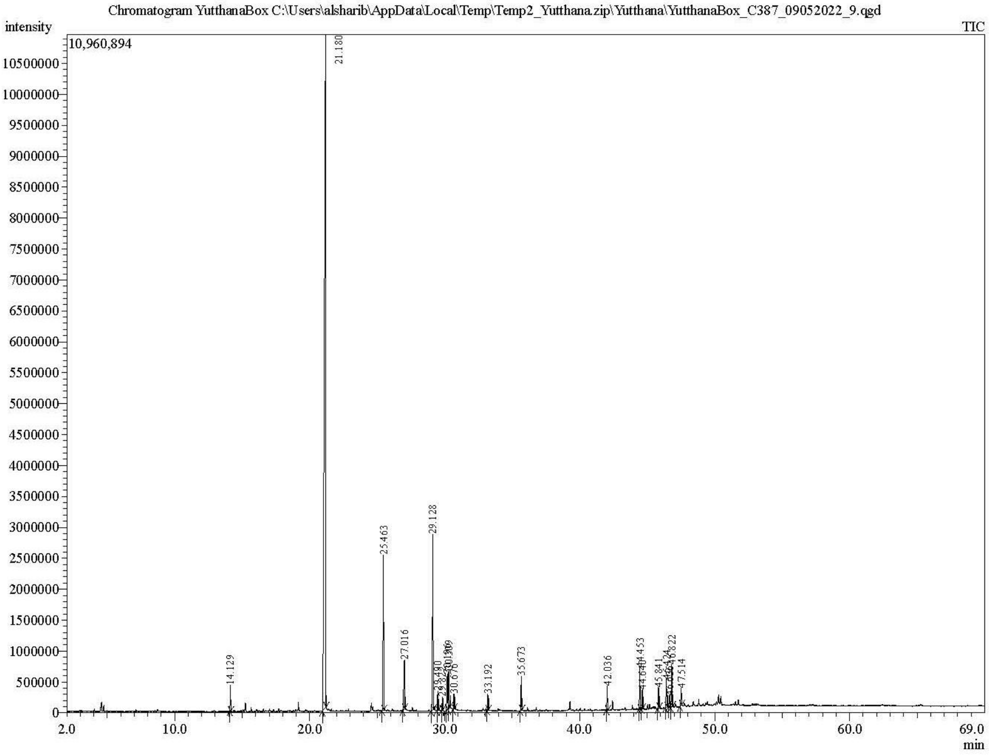

Peak Report TIC

Peak#	R.Time	I.Time	F.Time	Area	Area%	Height	Height%	Name
1	14.129	14.080	14.185	1048100	0.90	422472	1.79	Copaene
2	21.180	20.940	21.230	75403673	64.44	10878390	46.22	2-Cyclohexen-3-ol-1-one, 2-[11-phenylundeca
3	25.463	25.365	25.525	7560605	6.46	2505204	10.64	2-Cyclohexen-3-ol-1-one, 2-dodecanoyl-
4	27.016	26.885	27.075	3405742	2.91	797933	3.39	n-Hexadecanoic acid
5	29.128	29.015	29.185	9897100	8.46	2834886	12.04	
6	29.490	29.430	29.545	847965	0.72	316093	1.34	2-Cyclohexen-3-ol-1-one, 2-dodecanoyl-
7	29.822	29.770	29.880	588194	0.50	222784	0.95	Phytol
8	30.196	30.095	30.245	2514824	2.15	578345	2.46	Linoelaidic acid
9	30.309	30.245	30.410	2551168	2.18	618016	2.63	7-Tetradecenal, (Z)-
10	30.676	30.620	30.740	675233	0.58	248860	1.06	Octadecanoic acid
11	33.192	33.135	33.255	739777	0.63	261124	1.11	2-Cyclohexen-3-ol-1-one, 2-[11-phenylundeca
12	35.673	35.605	35.745	1606480	1.37	549997	2.34	(E)-5-(Benzo[d][1,3]dioxol-5-yl)-1-(piperidin-
13	42.036	41.975	42.105	1072833	0.92	393136	1.67	Tetratetracontane
14	44.453	44.380	44.520	1944763	1.66	675079	2.87	9,19-Cyclolanost-24-en-3-ol, (3.beta.)-
15	44.640	44.580	44.705	840849	0.72	304445	1.29	Hentriacontane
16	45.841	45.780	45.915	1063021	0.91	329741	1.40	Olean-12-en-28-al, cyclic 1,2-ethanediyl merca
17	46.424	46.345	46.500	1615605	1.38	456199	1.94	Ergost-5-en-3-ol, (3.beta.)-
18	46.636	46.590	46.695	501789	0.43	178807	0.76	Lanosta-8,24-dien-3-one
19	46.822	46.745	46.900	2186243	1.87	684715	2.91	Stigmasterol
20	47.514	47.445	47.585	946105	0.81	281379	1.20	.gamma.-Sitosterol
				117010069	100.00	23537605	100.00	

Figure S16 The GC/MS spectrum and reported data of *P. ranongensis (*c387) from the library.
